# Characterizing Differences in Functional Connectivity Between Posterior Cortical Atrophy and Semantic Dementia by Seed-Based Approach

**DOI:** 10.3389/fnagi.2022.850977

**Published:** 2022-04-29

**Authors:** Yi Chen, Qingze Zeng, Yunyun Wang, Xiao Luo, Yan Sun, Lumi Zhang, Xiaoyan Liu, Kaicheng Li, Minming Zhang, Guoping Peng

**Affiliations:** ^1^Department of Neurology, The First Affiliated Hospital, Zhejiang University School of Medicine, Hangzhou, China; ^2^Department of Radiology, The Second Affiliated Hospital, Zhejiang University School of Medicine, Hangzhou, China; ^3^Department of Neurology, The Affiliated Hospital of Hangzhou Normal University, Hangzhou, China; ^4^Department of Neurology, Shengzhou People’s Hospital, Shengzhou, China

**Keywords:** posterior cortical atrophy, semantic dementia, language deficits, functional connectivity, network

## Abstract

**Background:**

Posterior cortical atrophy (PCA) and semantic dementia (SD) are focal syndromes involving different cerebral regions. This study aimed to demonstrate the existence of abnormal functional connectivity (FC) with an affected network in PCA and SD.

**Methods:**

A total of 10 patients with PCA, 12 patients with SD, and 11 controls were recruited to undergo a detailed clinical history interview and physical examination, neuropsychological assessments, and PET/MRI scan. Seed-based FC analyses were conducted to construct FC in language network, visual network, and salience network. The two-sample *t*-test was performed to reveal distinct FC patterns in PCA and SD, and we further related the FC difference to cognition. Meanwhile, the uptake value of fluorodeoxyglucose in regions with FC alteration was also extracted for comparison.

**Results:**

We found a global cognitive impairment in patients with PCA and SD. The results of FC analyses showed that patients with PCA present decreased FC in left precentral gyrus to left V1 and increased FC in right inferior frontal gyrus to right V1 in the visual network, right medial frontal gyrus and left fusiform to left anterior temporal lobe and post-superior temporal gyrus in the language network, and left superior temporal gyrus to left anterior insula in the salience network, which were related to cognitive function. Patients with SD had decreased FC from right superior frontal gyrus, right middle frontal gyrus and right superior frontal gyrus to left anterior temporal lobe, or post-superior temporal gyrus in the language network, as well as left superior frontal gyrus to right anterior insula in the salience network, positively relating to cognitive function, but increased FC in the right superior temporal gyrus to left anterior temporal lobe in the language network, and right insula and left anterior cingulum to right anterior insula in the salience network, negatively relating to cognitive function. Most of the regions with FC change in patients with PCA and SD had abnormal metabolism simultaneously.

**Conclusion:**

Abnormal connectivity spread over the cortex involving language and salience networks was common in patients with PCA and SD, whereas FC change involving the visual network was unique to patients with PCA. The FC changes were matched for cognitive deficits.

## Introduction

Alzheimer’s disease (AD) is well known as the most common cause of dementia worldwide; however, there are increasingly other degenerative pathological brain processes that may cause dementia. Based on neuroradiology, focal degenerative dementia syndromes are classified by a lobar approach, which was associated with a characteristic clinical picture (Lam et al., 2013). For example, behavioral-variant frontotemporal dementia was localized in the frontal lobe, semantic dementia (SD) in the anterior temporal lobe (ATL), and posterior cortical atrophy (PCA) in the occipital-parietal-temporal lobe (Ossenkoppele et al., 2015; Pasquini et al., 2020). AD is the most common pathology of PCA, whereas Lewy body disease, corticobasal degeneration, and prion disease are pathologically less frequently associated (Holden et al., 2020); however, most cases of SD have TDP-43 pathology at post mortem, and a small minority are accounted for tauopathies and AD (Marshall et al., 2018). Reviewing the previous literature, the overlapping could be found in presentation and even the underlying dysfunction between these focal syndromes, such as PCA and SD.

Posterior cortical atrophy is characterized by predominant visuospatial and visuoperceptual dysfunction, which are selectively associated with primary visual (caudal), occipitoparietal (dorsal), occipitotemporal (ventral), and dominant parietal cerebral pathways bilaterally, with varying degrees of lateralization (Crutch et al., 2017; Miller et al., 2018; Firth et al., 2019). Regarding the category specificity in patients with PCA, their visual deficits received far more attention than their less obvious language, attention, and memory deficits (Shebani et al., 2017), whereas non-visual presentations can also be seen in PCA. However, there indeed existed language deficits in PCA compared to healthy. For example, Crutch et al. (2013) found that all the domains of language examined were impaired in PCA, especially naming and fluency. Simultaneously, Rik Ossenkoppele et al. showed that naming was significantly damaged in PCA compared with healthy while fluency was not. Both these studies have not been related to regions, which have already presented neuroimaging dysfunction. PCA also had difficulty in semantic categories with impairment in processing function words, number words, and prepositions, and the region of primary hypometabolism and atrophy in PCA might be the fundamental factors (Shebani et al., 2017). Although it has been concluded that the language functions have been, to some degree, damaged, there was no research reflecting the language network change in PCA.

The progressive loss of language abilities is the predominant neurobehavior of primary progressive aphasia with three main phenotypic clinical presentations, including semantic variants (SD), logopenic variants (lvPPA), and non-fluent/agrammatic variants (nfvPPA) (Ranasinghe et al., 2017). Each PPA variant has a unique anatomical pattern of neuronal loss, namely, asymmetric bilateral ATL atrophy in SD, left posterior temporoparietal atrophy in lvPPA, and left posterior frontoinsular and subcortical atrophy in nfvPPA (Mandelli et al., 2014; Leyton et al., 2016). Meanwhile, each PPA variant revealed a distinct deficit in language function with anomia, loss of word comprehension, object concepts and semantic representations in SD; prominent phonological impairments in lvPPA; and motor speech and/or grammatical processing in nfvPPA (Gorno-Tempini et al., 2011). Consistent with PCA, one of the primary language deficits was naming in patients with SD, and both had semantic word-processing problems. Anatomically, the regional lesions of SD encompassed a large portion of the temporal lobes and extended to posterior temporal, parietal cortex, frontal, anterior insular, and anterior cingulate (Collins et al., 2017). Previous neuroimaging studies have shown specific impacts within language networks in SD—taking the ATL as the hub region, distributing to nodes in frontal, posterior temporal, and even parietal areas. Based on this hub with distributed node framework, intrinsic functional connectivity (FC) integrity was disrupted in a wide array of selective regions. Salience network might be involved in this framework, which was linked to clinical anxiety and lack of self-regulation (Day et al., 2013), and this pattern seemed to be presented in PCA (Fredericks et al., 2019). In addition, patients with SD were not only confused by face recognition and emotional processing implemented by posterior cortices (Ding et al., 2020), and they are also expected to be more impaired in words carrying visual semantic information about how an object looks (e.g., its color and form) (Shebani et al., 2017). However, the node in the abnormal region of SD has not reported the relevance of the visual network.

Posterior cortical atrophy and SD were selected because they have well-defined focal lesions in the cortex. Areas affected in each condition are proposed to make a region-specific contribution to language and visual processing (Shebani et al., 2017). Resting-state functional magnetic resonance imaging (rs-fMRI) is one of the neuroimaging techniques that allows the investigation of functional brain networks (Montembeault et al., 2019). A growing body of rs-fMRI research suggests that abnormalities of these networks are associated with presentations of neurological disorders belonging to proteinopathies, such as AD (including PCA) and frontotemporal dementia (including SD) (Pini et al., 2021). With advances in neuroimaging models, it is now clear that the network is distributed (Ghosh, 2020). Pathogenic proteins spreading through large-scale networks produce macroscopic signatures of network dysfunction that might differentiate neurodegenerative diseases (Warren et al., 2013). Brain network FC is considered sensitive and may be a useful non-invasive marker for dementia (Spina et al., 2019).

The main hypotheses in this study are based on factors such as (1) specific regional brain lesions in the two patient groups and relevance of these regions to clinical manifestations; (2) previous studies demonstrating similar language deficits, visual semantic processing, and emotional disorder in the two patient groups; (3) the fact that PCA lacks language network research when SD lacks visual network research; and (4) the necessity of making comparisons about the pattern of network dysfunction on account of different pathology but similar symptoms. Therefore, we showed how FC changes in PCA and SD related to three networks, a “visual network” (atrophied in PCA), a “language network” (atrophied in SD), and a “salience network” (atrophied in SD and both have shown to be altered previously). Then, to explore the role of connectivity from seed to specific regions, we correlated connectivity intensity and neuropsychological features. Finally, we further compared the value of standard uptake value ratio (SUVR) in the specific regions with connectivity altering.

## Materials and Methods

### Participants

A total of 11 patients with PCA, 14 patients with SD, and 11 matched controls were recruited from the Memory Clinic and Neurology Unit, First Affiliated Hospital of Zhejiang University School of Medicine, Hangzhou, China. All participants underwent a detailed clinical history interview and physical examination, neuropsychological assessment, and a (positron emission tomography) PET/MRI scan. All participants were native Chinese speakers, right-handed, and provided written informed consent. This study was approved by the Institutional Ethics Board of the First Affiliated Hospital, Zhejiang University School of Medicine.

#### Patients With PCA

Participants with PCA fulfilled previously proposed clinical diagnostic criteria (Crutch et al., 2017). Core features included clinical features (insidious onset, gradual progression, and prominent early disturbance of visual functions); cognitive features [space perception deficit, simultanagnosia, object perception deficit, constructional dyspraxia, environmental agnosia, oculomotor apraxia, dressing apraxia, optic ataxia, alexia, left/right disorientation, acalculia, limb apraxia (not limb-kinetic), apperceptive prosopagnosia, agraphia, homonymous visual field defect, and finger agnosia]—at least three of the following must be present as early or presenting features, with evidences of their impact on activities of daily living, and relatively spared anterograde memory function, speech and non-visual language functions, executive functions and behavior and personality must be evident; neuroimaging features [predominant occipitoparietal or occipitotemporal atrophy/hypometabolism MRI/FDG-PET (2-fluoro-2-deoxy-D-glucose FDG)]; exclusion criteria—evidence of a brain tumor or other mass lesion sufficient to explain the symptoms, significant vascular diseases, including focal stroke sufficient to explain the symptoms, afferent visual cause (e.g., optic nerve, chiasm, or tract), and other identifiable causes for cognitive impairment (e.g., renal failure). All the patients with PCA were fulfilled with pure-PCA.

#### Patients With SD

Patients with SD had normal or corrected-to-normal hearing and vision, and no history of alcoholism, head trauma, and psychiatric or other neurological illness. The neuropsychological performance and predominant ATL atrophy of each patient met the diagnostic criteria for SD (Gorno-Tempini et al., 2011). For clinical diagnosis of SD, both of the following core features must be present, namely, (1) impaired confrontation naming and (2) impaired single-word comprehension. In addition, at least three of the following other diagnostic features must be present, namely, (1) impaired object knowledge, particular for low-frequency or low-familiarity items, (2) surface dyslexia or dysgraphia, (3) spared repetition, and (4) spared speech production (grammar and motor speech). Imaging-supported SD, including both of the following criteria, must be present, namely, (1) clinical diagnosis of SD and (2) imaging must show one or more of the following results (a, predominant ATL atrophy; b, predominant ATL hypoperfusion or hypometabolism on SPECT or PET).

#### Healthy Control Subjects

Healthy control subjects also had normal or corrected-to-normal hearing and vision, and no history of alcoholism, head trauma, and psychiatric or neurological illness.

### Neuropsychological Assessment

All participants underwent a standardized neuropsychological battery within 7 days of imaging to evaluate cognitive functioning, consisting of assessments of global functioning and language functioning. Patients (PCA or SD groups) and HC groups were compared in each of these domains. Global functioning was measured using the Mini-Mental State Exam (MMSE), Montreal Cognitive Assessment (MoCA), clinical dementia rate (CDR), and Clock Drawing Test (CDT). Language function was evaluated by the Boston Naming Test (BNT) scores.

### Imaging Data Collection

All images were performed on a hybrid 3.0-Tesla TOF PET/MRI system (Signa, GE Healthcare), with PET and MR images simultaneously acquired in 19-channel head and neck union coil. Each patient was required to fast for at least 6 h and then received a manual intravenous injection of ^18^F-FDG (0.08–0.12 mCi/kg). Then, 45 min after this injection, the patients were placed in the PET/MR scanner, instructed to remain calm in a supine position, and with their eyes closed. All images were completed after 30 min. For 3D BRAVO T1-weighted sequence, the following parameters were applied, namely, echo time (TE) = 3.2 ms; repetition time (TR) = 8.5 ms; 170 sagittal slices; inversion time (TI) = 400 ms; within-plane field of view (FOV) = 256 mm × 256 mm; flip angle = 15°; voxel size = 1 mm × 1 mm × 1 mm; and bandwidth = 240 Hz/pix. For blood oxygenation level-dependent resting-state functional MRI, the following parameters were applied, namely, time point = 200; TR = 2,000 ms; TE = 35 ms; flip angle = 90°; 32 sagittal slices; FOV = 220 mm × 220 mm; and matrices = 64 × 64.

### Imaging Data Preprocessing

#### Functional MRI

The resting-state functional MRI (rsfMRI) data preprocessing was performed using the resting-state fMRI toolbox (DPARSF) (Yan et al., 2016) based on the Statistical Parametric Mapping 12 (SPM12) on the MATLAB platform (MathWorks, Natick, MA, United States). One patient with PCA was excluded because of incomplete images. The first 10 rsfMRI scans were discarded for the signal equilibrium and subject’s adaptation to the scanning noise. The remaining 190 images were corrected for timing differences in slice acquisition. Then, head motion correction was performed. Subjects with more than 3 mm maximum displacement in any of the x, y, or z directions or 3° of any angular motion were discarded. Two participants (SD) did not meet these criteria and were excluded from the initial sample. Then, the rsfMRI data based on rigid-body transformation were subsequently normalized to a Montreal Neurological Institute space using the echo-planar images template (Calhoun et al., 2017) and then resampled into 3 mm × 3 mm × 3 mm cubic voxel. Functional images were spatially smoothed with a 6 mm × 6 mm × 6 mm Gaussian kernel of full width at half maximum to decrease spatial noise. Linear trends estimation was finally performed.

#### Structural MRI

The T1 images were preprocessed by using the CAT12 toolbox^1^ in SPM12 (Wellcome Trust Centre for Neuroimaging^2^) (Friston et al., 1995) and was performed on MATLAB software (MathWorks, Natick, MA, United States). Images preprocessing was performed by the CAT12 toolbox under the default setting. First, all 3D T1-weighted MRI scans were normalized using an affine followed by non-linear registration, corrected for bias field inhomogeneities, and then segmented into gray matter (GM), white matter (WM), and cerebrospinal fluid (CSF) components (Ashburner and Friston, 2005). Next, the segmented scans were normalized into a standard MNI space by using the Diffeomorphic Anatomic Registration Through Exponentiated Lie algebra algorithm (Ashburner, 2007), which can provide more precise spatial normalization to the template.

#### FDG-PET Data Preprocessing

The PETPVE12 toolbox (PETPVE12: an SPM toolbox for PVE correction in brain PET, application to amyloid imaging with FDG-PET) was used to analyze PET data. First, the structural MRI (T1-weighted) data were segmented into GM, WM, CSF, and skull-stripped image based on the segmentation function of the VBM8 toolbox. Second, the structural MRI (without skull stripping) was used as “reference” images, and FDG-PET images were used as “source image.” Third, a voxel-based method was performed using the three-compartmental algorithm, including GM, WM, and CSF, which is described as Muller-Gartner et al. (1992) (MG) (Muller-Gartner et al., 1992) or “modified Müller-Gärtner” (mMG) (Rousset et al., 1998) to correct the PVE of the PET images.

### Seed-Based FC

Seed regions of interest (ROIs) were based on previous studies using the DynamicBC toolbox (Liao et al., 2014). On account of the changing mode of connectivity that was reported in the two diseases, seeds were selected based on previous studies for primary visual network [left V1 (−11, −81, 7) and right V1 (11, −78, 9)] (Glick-Shames et al., 2019), language network [left ATL (39, 15, −33)and left posterior superior temporal gyrus (−49, −9, 6)] (Migliaccio et al., 2016; Montembeault et al., 2019), and salience network [left anterior insula (−32, 24, −6) and right anterior insula (37, 25, −4)] (Fredericks et al., 2019). The seed regions were 4-mm radius centered at the peak. For each seed, correlations were calculated between the averaged signal from the seed and each individual ROI across the brain to configure a statistical map.

### Statistical Analysis

Quantitative variables are expressed as the mean and standard deviation except gender. The categorical variables are given as absolute and relative frequencies. All statistical analyses were performed using the IBM SPSS26.0 statistical software for Windows. Regarding the demographics, the chi-square test was used for gender distribution difference assessment (*p* < 0.05). We then used the analysis of two-sample *t*-test to compare the education, age, and neuropsychological scales among all groups.

Voxel-wise statistical analyses of FC, GM, and FDG-PET SUVR were conducted by DPABI toolbox. The effect of group on FC was assessed using the two-sample *t*-test, controlled for age, gender, and years of education. Comparison of FC maps was restricted to the GM areas, and the Gaussian random field (GRF) method was applied to multiple-comparison correction. The statistical threshold was set at *p* < 0.005 with a cluster-level *p* < 0.05 (two-tailed). To test the clinical significance, we also correlated mean FC values from the clusters with neuropsychological scales by partial correlation analysis, controlled for age, gender, years of education, and FC values in seed ROIs. The relationship between FC values and cognitive test scores is presented as a scatter diagram drawn using GraphPad Prism 8.0. The ROI with FC discrepancy was used as template to extract the SUVR of FDG-PET and then compared between the two groups using two-sample *t*-test.

## Results

### Demographic and Clinical Characteristics

There were no statistically significant differences in gender, age, or education between patients (PCA or SD) and controls (*p* > 0.05), except that patients with SD were older than controls (*p* = 0.017). The age comparison between PCA and SD was also significant (*p* = 0.019). There were substantial differences in neuropsychological scores between patients (PCA or SD) and controls (*p* < 0.001). Detailed information can be found in Table 1. Patients with PCA and SD performed worse across global cognitive function, BST, and CDT tests. Patients with PCA performed significantly worse than SD in neuropsychological CDT scores (*p* = 0.001), whereas other neuropsychological tests had no significant differences between the two groups.

**TABLE 1:** Demographic and neuropsychological data.

Demographic characteristics	NC *N* = 11	PCA *N* = 10	SD *N* = 12	PCA vs NC *P* value	SD vs NC *P* value	PCA vs SD *P* value
Sex M:F	3:8	6:4	4:8	0.291	0.100	0.391
Age (years)	60.00 ± 6.387	59.70 ± 6.865	66.67 ± 5.944	0.918	0.017	0.019
Education (years)	8.82 ± 1.991	10.20 ± 4.756	6.50 ± 3.826	0.588	0.094	0.057
MMSE	25.64 ± 2.541	15.30 ± 5.376	14.33 ± 6.329	< 0.001	<0.001	0.707
MoCA	23.45 ± 2.252	9.90 ± 5.216	10.08 ± 4.833	< 0.001	<0.001	0.933
CDR	0.045 ± 0.1508	1.800 ± 0.6325	1.333 ± 0.6155	< 0.001	<0.001	0.096
BNT	22.82 ± 1.662	13.50 ± 4.927	10.42 ± 5.178	< 0.001	<0.001	0.171
CDT	4.00 ± 0.000	0.80 ± 0.632	2.25 ± 1.215	< 0.001	<0.001	0.003

*Data are presented as means ± standard deviations except for sex (male to female ratio).*

*NC, normal control; PCA, posterior cortical atrophy; SD, semantic dementia; MMSE, Mini-Mental State Examination; MoCA, Montreal Cognitive Assessment; CDR, clinical dementia rating; BNT, Boston Naming Test; CDT, Clock Drawing Test.*

### Seed-Based FC

**FIGURE 1:**
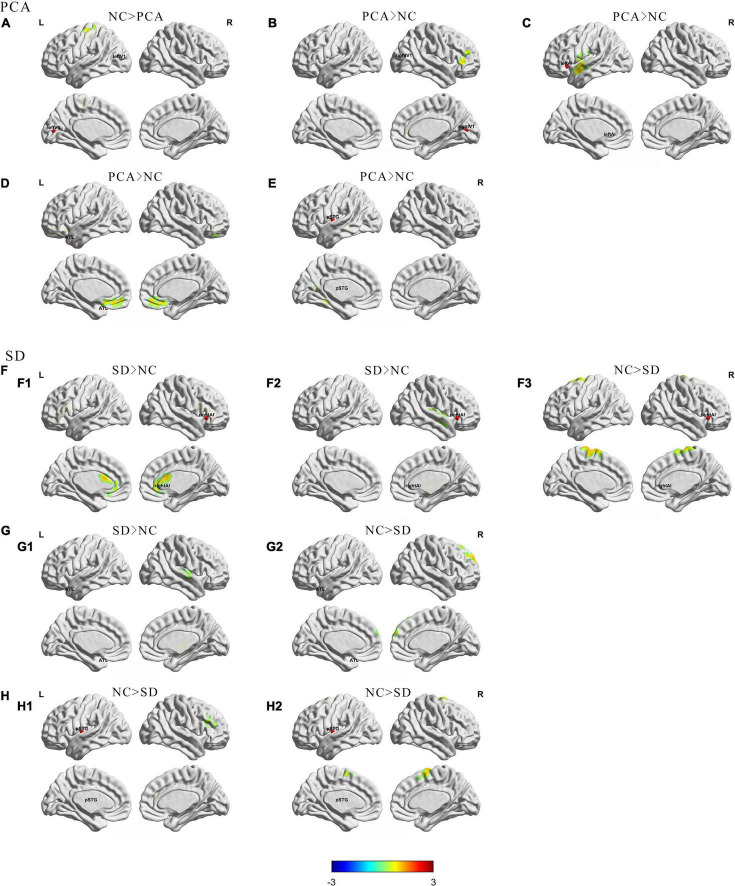
Group differences in regional functional connectivity from seed between patients (PCA or SD) and controls. The results were mapped on the brain surface using BrainNet Viewer (Xia et al., 2013) showing reduced or increased regional functional connectivity from seeds **(A)** left V1; **(B)** right V1; **(C)** left anterior insula; **(D,G)** left anterior temporal lobe; **(F)** right anterior insula; **(E,H)** left superior temporal gyrus) in PCA compared with NC **(A–E)** or SD **(F–H)** compared with NC (two-sample *t*-test, voxel level *p* < 0.005, cluster level *p* < 0.05, GRF corrected). The red spheres were localizations of seeds. Colors indicate *t* scores. ATL, anterior temporal lobe; pSTG, post-superior temporal gyrus; AI, anterior insula.

First, to compare with controls, taking the left V1 region in visual network as seed, PCA showed decreased FC between this region and left precentral gyrus (Figure 1A). Meanwhile, the FC was significantly increased between the right V1 seed and the right inferior frontal gyrus (Figure 1B). Taking the left ATL in language network as seed, patients with PCA showed increased FC to right medial frontal gyrus (Figure 1D). Left post superior temporal gyrus, as another seed in the language network, showed increased FC from this region to left fusiform in patients with PCA (Figure 1E). Increased FC was also significant between left anterior insula SN seed to left superior temporal gyrus (Figure 1C).

Second, compared with controls, changes in the SD group are more focused on the temporal lobe. Decreased FC has been showed from the left ATL seed in the language network to the right superior frontal gyrus (Figure 1G2), as well as another language seed post-superior temporal gyrus to right middle frontal gyrus and right superior temporal gyrus (Figure 1H), whereas increased FC was showed from left ATL to the right superior temporal gyrus (Figure 1G1). Interestingly, taking the anterior insula in the salience network as the seed, patients with SD showed increased FC from right side to right insula and left anterior cingulum, and decreased FC from right seed to left superior frontal gyrus. However, the left seed did not have significant change (Figure 1F). Besides, left and right V1 regions in visual network did not have significant FC change in patients with SD either. Detailed information can be found in Tables 2, 3.

**TABLE 2:** Regions of changed resting state functional connectivity in patients with PCA compared with healthy controls.

Seeds	Clusters					
	
	Peak regions	x	y	Z	Number of voxels	Peak intensity
Left anterior temporal lobe	Right medial frontal gyrus	9	45	−15	131/232	5.3305
Left anterior insula	Left superior temporal gyrus	−57	−3	0	111/231	5.7633
Left superior temporal gyrus	Left fusiform	−33	−19	−15	74/188	5.318
Left V1	Left precentral areas	−36	−24	60	71/169	–6.5434
Right V1	Right inferior frontal gyrus	57	39	15	93/174	5.0356

*Coordinates (x, y, z) are in Montreal Neurological Institute space.*

*Results are shown at p < 0.05 GRF corrected.*

**TABLE 3:** Regions of changed resting state functional connectivity in patients with SD compared with healthy controls.

Seeds	Clusters					
	
	Peak regions	x	Y	z	Number of voxels	Peak intensity
Left anterior temporal lobe	Right superior frontal gyrus	−2	63	36	158/225	–6.0639
	Right superior temporal gyrus	39	−21	−3	60/138	6.3062
right anterior insula	Right insula	45	−3	−9	59/158	4.9877
	Left superior frontal gyrus	−3	−15	72	141/382	–6.44
	Left anterior cingulum	0	18	21	70/186	7.176
left superior temporal gyrus	Left middle frontal gyrus	27	30	33	70/105	–5.6875
	Right superior frontal gyrus	9	−3	72	91/145	–5.7519

*Coordinates (x, y, z) are in Montreal Neurological Institute space.*

*Results are shown at p < 0.05 GRF corrected.*

The comparisons between PCA and SD have found that PCA showed decreased FC from left ATL to bilateral middle occipital gyrus, as well as from right V1 to bilateral anterior cingulum (Supplementary Figure 1).

### Relationship Between FC and Neuropsychological Performance

**FIGURE 2:**
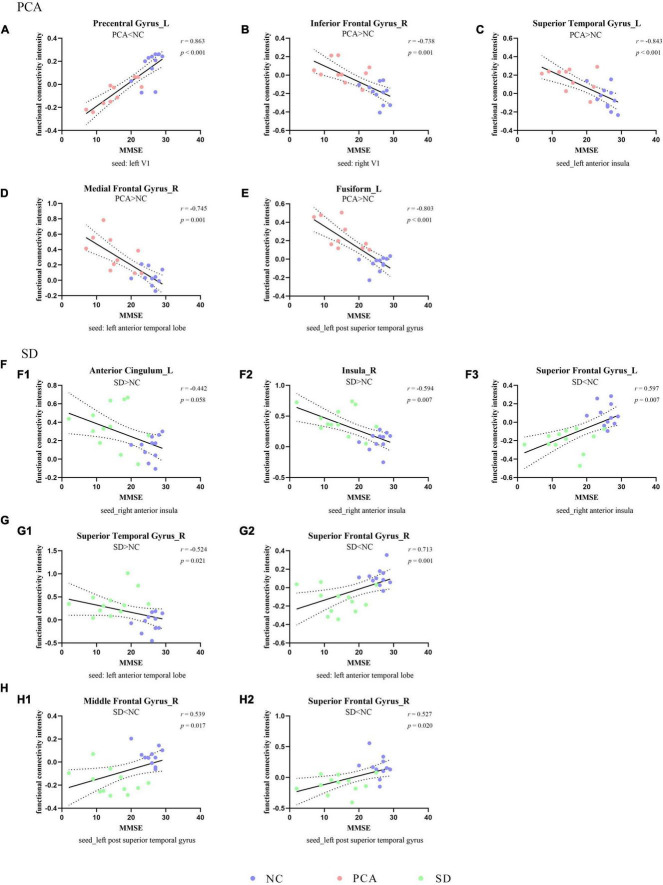
Correlation between functional connectivity and global cognition. The scatterplots illustrate the associations between MMSE scores and intensity of functional connectivity in patients [PCA (A–E) or SD (F–H)] and controls based on different seeds [**(A)** left V1, **(B)** right V1, **(C)** left anterior insula, **(D,G)** left anterior temporal lobe, **(F)** right anterior insula, and **(E,H)** left superior temporal gyrus].

**FIGURE 3:**
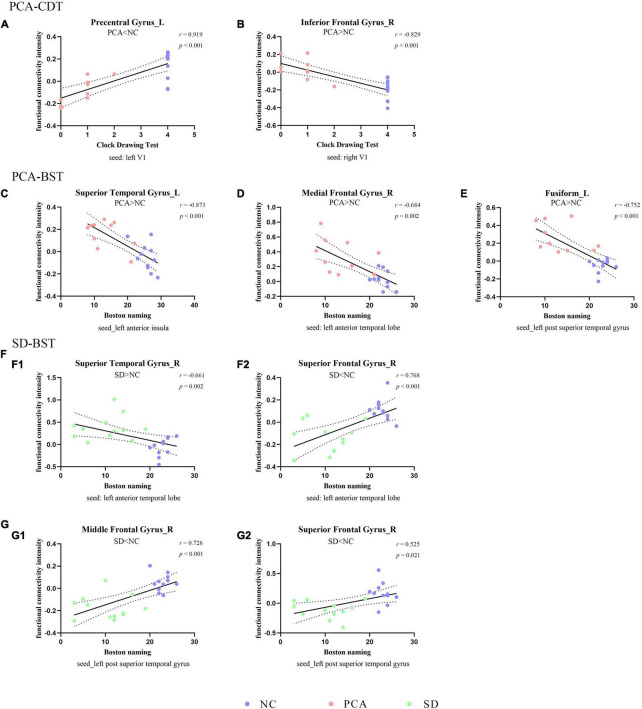
Correlation between functional connectivity and subdomain of cognition. The scatterplots illustrate the associations between CDT **(A,B)** or BST **(C–G)** and intensity of functional connectivity in patients (PCA: **A–E** or SD: **F–G**) and controls based on different seeds [**(A)** left V1, **(B)** right V1, **(C)** left anterior insula, **(D,F)** left anterior temporal lobe, and **(E,G)** left superior temporal gyrus].

The global two groups (one patient group and controls) showed significant and strong correlations between neuropsychological measures and regional mean FC intensity. All the regional mean FCs were significant in the relative two groups. Positive correlations were found with regions showing diminished FC from seed, and negative correlations were found with regions showing increased FC from seed. However, the patient group alone was hardly seen significant correlations between neuropsychological measures and regional mean FC intensity both in PCA and SD, and the significant relations were only in PCA. Detailed information can be found in Figures 2, 3, Supplementary Figure 2, Table 4, and Supplementary Table 1.

**TABLE 4:** Partial correlation analysis between functional connectivity intensity and neuropsychological scales.

Functional connectivity changing groups	Clusters (seeds)	Neuropsychological scales			
		
		MMSE	MoCA	BST	CDT
PCA compared to NC	Right medial frontal gyrus (left anterior temporal lobe) PCA > NC	*r* = −0.745, *p* = 0.001	*r* = −0.826, *p* < 0.001	*r* = −0.684, *p* = 0.002	*r* = −0.824, *p* < 0.001
	Left superior temporal gyrus (left anterior insula) PCA > NC	*r* = −0.843, *p* < 0.001	*r* = −0.867, *p* < 0.001	*r* = −0.873, *p* < 0.001	*r* = −0.890, *p* < 0.001
	Left fusiform (left superior temporal gyrus) PCA > NC	*r* = −0.803, *p* < 0.001	*r* = −0.794, *p* < 0.001	*r* = −0.752, *p* < 0.001	*r* = −0.831, *p* < 0.001
	Left precentral areas (left V1) PCA < NC	*r* = 0.863, *p* < 0.001	*r* = 0.922, *p* < 0.001	*r* = 0.892, *p* < 0.001	*r* = 0.919, *p* < 0.001
	Right inferior frontal gyrus (right V1) PCA > NC	*r* = −0.738, *p* = 0.001	*r* = −0.824, *p* < 0.001	*r* = −0.780, *p* < 0.001	*r* = −0.829, *p* < 0.001
SD compared to NC	Right superior frontal gyrus (left anterior temporal lobe) SD < NC	*r* = 0.713, *p* = 0.001	*r* = 0.771, *p* < 0.001	*r* = 0.768, *p* < 0.001	*r* = 0.648, *p* = 0.003
	Right superior temporal gyrus (left anterior temporal lobe) SD > NC	*r* = −0.524, *p* = 0.021	*r* = −0.642, *p* = 0.003	*r* = −0.661, *p* = 0.002	*r* = −0.518, *p* = 0.023
	Right insula (right anterior insula) SD > NC	*r* = −0.594, *p* = 0.007	*r* = −0.661, *p* = 0.002	*r* = −0.651, *p* = 0.003	*r* = −0.627, *p* = 0.004
	Left superior frontal gyrus (right anterior insula) SD < NC	*r* = 0.597, *p* = 0.007	*r* = 0.659, *p* = 0.002	*r* = 0.699, *p* = 0.001	*r* = 0.590, *p* = 0.008
	Left anterior cingulum (right anterior insula) SD > NC	*r* = −0.442, *p* = 0.058	*r* = −0.525, *p* = 0.021	*r* = −0.492, *p* = 0.032	*r* = −0.483, *p* = 0.036
	Right middle frontal gyrus (left superior temporal gyrus) SD < NC	*r* = 0.539, *p* = 0.017	*r* = 0.611, *p* = 0.005	*r* = 0.726, *p* < 0.001	*r* = 0.535, *p* = 0.018
	Right superior frontal gyrus (left superior temporal gyrus) SD < NC	*r* = 0.527, *p* = 0.020	*r* = 0.544, *p* = 0.016	*r* = 0.525, *p* = 0.021	*r* = 0.541, *p* = 0.017

*NC, normal control; PCA, posterior cortical atrophy; SD, semantic dementia; MMSE, Mini-Mental State Examination; MoCA, Montreal Cognitive Assessment; BNT, Boston Naming Test; CDT, Clock Drawing Test.*

### The Patterns of Atrophy and FDG-PET in Patients With PCA and SD

The patients with PCA shared a large region of brain atrophy mainly including bilateral associative parietooccipital, temporal regions, precuneus, and posterior cingulate, extending to frontal regions (Supplementary Figure 3). Patients with SD showed brain atrophy mainly in bilateral temporal, insula, and frontal regions (Supplementary Figure 3). Compared to patients with SD, the atrophy of PCA was more severe in left temporal regions (Supplementary Figure 3).

Similarly, patients with PCA showed hypometabolism mainly in bilateral parietal, occipital, temporal regions and precuneus, with extension to a part of frontal regions; hypometabolism in SD was primarily distributed in bilateral temporal and frontal areas (Supplementary Figure 4). To compare PCA with SD, patients with PCA showed that hypometabolism was in bilateral parietal, occipital, and left temporal lobe, and hypermetabolism was in the right temporal and bilateral frontal lobe (Supplementary Figure 4). In contrast to controls, based on regions with FC abnormality, patients with PCA showed hypometabolism in the left superior temporal gyrus, fusiform, superior frontal gyrus, middle frontal gyrus, and precentral areas, as well as right inferior frontal gyrus, superior temporal gyrus, and insula (*p* < 0.05). Meanwhile, patients with SD showed hypometabolism in the left temporal superior gyrus, superior frontal gyrus, and right frontal superior gyrus, right insula (*p* < 0.05). Detailed information can be found in Table 5.

**TABLE 5:** Averaged ^18^F-FDG SUVR in cluster with abnormal connectivity to seed in normal controls and patients with PCA and SD.

Functional connectivity changing groups	Clusters (seeds)	SUVR value			*P* value		
			
		NC	PCA	SD	PCA vs NC	SD vs NC	PCA vs SD
PCA compared to NC	Right medial frontal gyrus (left anterior temporal lobe) PCA > NC	0.66 ± 0.09	0.67 ± 0.07	0.49 ± 0.05	0.757	0.971	< 0.001
	Left superior temporal gyrus (left anterior insula) PCA > NC	0.56 ± 0.08	0.43 ± 0.10	0.45 ± 0.11	0.003	0.009	0.726
	Left fusiform (left superior temporal gyrus) PCA > NC	0.12 ± 0.06	0.01 ± 0.05	0.05 ± 0.09	< 0.001	0.029	0.199
	Left precentral areas (left V1) PCA < NC	0.59 ± 0.08	0.44 ± 0.12	0.59 ± 0.12	0.004	0.999	0.011
	Right inferior frontal gyrus (right V1) PCA > NC	0.49 ± 0.09	0.39 ± 0.06	0.40 ± 0.16	0.010	0.105	0.944
SD compared to NC	Right superior frontal gyrus (left anterior temporal lobe) SD < NC	0.61 ± 0.09	0.53 ± 0.13	0.46 ± 0.12	0.148	0.005	0.234
	Right superior temporal gyrus (left anterior temporal lobe) SD > NC	0.53 ± 0.10	0.45 ± 0.09	0.40 ± 0.11	0.08	0.01	0.279
	Right insula (right anterior insula) SD > NC	0.40 ± 0.10	0.22 ± 0.08	0.94 ± 0.18	0.091	< 0.001	0.003
	Left superior frontal gyrus (right anterior insula) SD < NC	0.60 ± 0.09	0.48 ± 0.13	0.37 ± 0.14	0.023	< 0.001	0.059
	Left anterior cingulum (right anterior insula) SD > NC	0.45 ± 0.15	0.39 ± 0.15	0.46 ± 0.17	0.345	0.920	0.316
	Left middle frontal gyrus (left superior temporal gyrus) SD < NC	0.66 ± 0.10	0.49 ± 0.10	0.61 ± 0.17	0.001	0.414	0.083
	Right superior frontal gyrus (left superior temporal gyrus) SD < NC	0.57 ± 0.15	0.48 ± 0.16	0.55 ± 0.18	0.203	0.777	0.353

*SUVR values are presented as means ± standard deviations.*

*NC, normal control; PCA, posterior cortical atrophy; SD, semantic dementia.*

## Discussion

We examined the characteristics involving the visual, language, and salience network in patients with SD or PCA. The major findings of this study are (1) patients with PCA would be impaired in language functions, such as naming; (2) patients with PCA have performed functional impairment involving visual network, while patients with SD have not; (3) patients with PCA and SD showed FC change from seed in language network and salience network to other regions, and the patterns of SD were more diffused than PCA; (4) hyperconnectivity, in general, was the main pattern in patients with PCA, whereas the hypo- and hyperconnectivity were level pegging in patients with SD; and (5) the hypometabolism and atrophy were disease-specific patterns in patients with PCA and SD widespread in posterior area of PCA but restricted in the temporal-frontal area of SD.

We found that PCA had language declining alike SD. Although the consensus required relatively spared speech and non-visual language functions, it emphasized the contrast between the posterior cortical dysfunction and the relative sparing of other cognitive domains, aiming to distinguish PCA from lvPPA (Crutch et al., 2017). Crutch et al. (2013) concluded that PCA was characterized by a progressive oral language dysfunction such as degradation of vision, literacy, and numeracy, and had prominent difficulties in word retrieval. Additionally, previous studies verified that patients with PCA would be impaired in semantic word processing, and spatial preposition disposing was even worse than SD, which could be due to the dysfunction of posterior regions in PCA (Shebani et al., 2017). It indicated that the specific regions of PCA are critical for word processing. A case-control study showed that most patients with PCA had non-language domain learning disabilities, whereas 27.8% presented with language-only learning disabilities (Miller et al., 2018). Therefore, despite the prominent deficits in visual domain, language damage exists in PCA. Characterizing and quantifying the aphasia associated with PCA is vital for clarifying differential diagnoses for clinical and research studies.

Consistent with the study that used bilateral V1 region as seeds (Glick-Shames et al., 2019), FC deficits in our PCA cohort were also observed compared to healthy controls. The previous study has seen reduced connections between V1 and higher visual areas, as well as between V1 and the frontal eye field (precentral gyrus) (Glick-Shames et al., 2019). Likewise, our study also showed reduced connections between left V1 and left precentral areas, known to be involved in control of eye movement and visual attention, which might account for the attentional/frontal deficits of this clinical syndrome. It seems that our FC results in the PCA suggest more localized damage. It is important to note that the seeds used in our study are based on a previous study (Glick-Shames et al., 2019). However, the patient cohort differed between our study and previous study in various aspects. The aberrant communication in response to brain damage and neural resilience during the disease stage is heterogeneity in diverse patient groups (Agosta et al., 2018). Then, possibly, the central regions, either within or between networks, are interconnected flexibly and the change may not synchronize (Pini et al., 2021). Further studies using longitudinal data are required to better understand the origin and spread of FC changes in PCA.

Besides, relative to controls, increased connectivity was apparent in our seed-voxel analysis showing intra- or inter-hemisphere of patients with PCA. Apart from right V1 and right inferior frontal gyrus, left ATL and right medial frontal gyrus, left superior temporal gyrus and left fusiform, as well as anterior insula and left superior temporal region have all seen FC increasing, and these increased FC intensities were negatively correlated to cognition. Although the anterior regions have not atrophied, the brain FC involves language and salience network. It has been reported that patients with PCA showed increased connectivity between key nodes of SN than controls, which is thought to play a role in social-emotional functioning (Fredericks et al., 2019). We first used seeds in language network to explore the brain FC change in patients with PCA, and its negative correlations to global cognitive scores and Boston Naming scores could explain the language deficits in PCA.

As expected, there were no significant differences in patients with SD compared to controls when using bilateral V1 region as seeds. However, seeds in language and salience networks have shown more regions that had FC change. It is worth emphasizing that ATL is the semantic “hub” and principle of semantic topography, which employs structure and connectivity of frontotemporal cortex (Pulvermuller et al., 2010). Hence, a series of studies used ATL as the seed to do the connectivity research (Guo et al., 2013; Wilson et al., 2014; Montembeault et al., 2019); one of these studies presented decreased FC between the left ATL and the cluster, including left post middle temporal gyrus, right medial orbitofrontal cortex and right medial superior frontal gyrus (Montembeault et al., 2019). In accordance with the study, our SD cohorts showed reduced connectivity between the left ATL and right superior frontal gyrus. Although it is not a critical language region, this region has been consistently reported as functionally connected to key regions of the language network (Hurley et al., 2015). Additionally, our study also observed decreased FC between the left superior temporal gyrus and right superior frontal gyrus. It indicated that right superior frontal gyrus might be essential to SD.

Few studies focused on salience network in patients with SD. Day et al. (2013) found that low-frequency fluctuations in the insula could predict behavioral changes in patients with frontotemporal dementia, including SD. We initially used anterior insula in the salience network as the seed, finding that connection from seed to right insula showed increased FC. It reminded that the intrinsic connectivity was increased within salience network in SD. Moreover, salience network FC is a critical neurological mechanism underlying interpersonal warmth (Toller et al., 2019). How the specific circuit of network works in patients with SD requires further research.

Except for diminished FC, there were increased brain FC in patients with PCA and SD, inversely correlated to cognition. The FC seeded by brain regions was vulnerable to any distinct neurodegenerative diseases, and the regions with higher connectional flow revealed greater disease-related vulnerability (Zhou et al., 2012). Neuroimmune may be a potential etiopathogenesis of dementia as the cascade of immune responses can occur as long as there is a neuronal injury or a potent immune stimulation (Cacabelos et al., 2016). A recent study showed how to link the FC with neuroinflammation with the combination of [^11^C]PK11195 PET and resting-state functional MRI. The results showed that the region with higher [^11^C]PK11195 binding values in individuals also showed increased connectivity between the default mode network, hippocampus, and other subcortical regions, mediating cognitive deficits in AD cohorts (Passamonti et al., 2019). Therefore, the change in increased FC may become a strong risk factor for PCA and SD.

^18^F-FDG-PET, as a potential sensitive molecular imaging marker indicating neuronal damage, is useful in subtyping dementia and helpful in managing patients, especially patients with early-onset dementia (Mukku et al., 2019). Both PCA and SD belong to early-onset dementia. With these two dementias characterized by the presence of disease-specific protein aggregates in and around neuronal cells, the neuroimaging-derived indexes may potentially reveal distinct neuropathological processes. Increasing evidence supports that before protein aggregates spread in preexisting networks they developed in topographic patterns (Seeley et al., 2009; Hodges et al., 2010; Bejanin et al., 2019). These neuropathological lesions were partly reflected in GM atrophy and hypometabolism (Bejanin et al., 2019). We compared the topographic discrepancies based on FC change regions in PCA and SD, showing that PCA has a more extended pattern of hypometabolism while SD is restricted in regions of temporal-frontal area. The results were consistent with the pattern of atrophy in our findings and previous studies (Montembeault et al., 2018; Duignan et al., 2021). Altogether, the disease-specific pattern might be suggested, whereas region-specific pattern might also be reflected as an obvious overlapping area in temporal lobe (e.g., language modality).

There are some limitations to this study. The size of our PCA and SD groups was relatively small, which limited the statistical power of our analyses. Consequently, the significant effects of these two groups might have been overestimated and need replication by an independent, ideally larger sample of PCA and SD. Second, the consistency between the connectivity results and the symptomatology suggested that the results are valuable for further studies. More specific language and semantic abilities (visual or non-visual) were not investigated. Future studies are required with more fine-grained tasks. One methodological limitation was that we just used FC to measure the topological attributes of the network.

## Conclusion

Based on global cognition degeneration, language deficits existed in both PCA and SD groups. The finding that patients with PCA had abnormal connectivity spreading over the cortex involving visual, language, and salience network also matched the observation that they showed several cognitive deficits. In contrast, the aberrant connectivity in patients with SD was restricted to frontal and temporal lobes, relating to the symptomatology. This study demonstrates that the progression of PCA and SD is determined not only by GM atrophy localization and distinct symptomatology, but also by the connectivity of the functional network. In general, regarding focal syndrome, the FC change involving visual network was specific to PCA, whereas SD was restricted to temporal and frontal areas.

## Data Availability Statement

The original contributions presented in the study are included in the article/Supplementary Material, further inquiries can be directed to the corresponding author/s.

## Ethics Statement

The studies involving human participants were reviewed and approved by the First Affiliated Hospital, Zhejiang University School of Medicine. The patients/participants provided their written informed consent to participate in this study.

## Author Contributions

YC collected the data, designed the study, and wrote the first draft of the article. QZ analyzed the MRI data and wrote the protocol. XL and KL assisted with study design and interpretation of findings. YW, YS, LZ, and XL collected clinical and MRI data. GP and MZ revised the manuscript for content, analysis and interpretation of data, and accept responsibility for conduct of research and final approval. All authors have contributed to and approved the final article.

## Conflict of Interest

The authors declare that the research was conducted in the absence of any commercial or financial relationships that could be construed as a potential conflict of interest.

## Publisher’s Note

All claims expressed in this article are solely those of the authors and do not necessarily represent those of their affiliated organizations, or those of the publisher, the editors and the reviewers. Any product that may be evaluated in this article, or claim that may be made by its manufacturer, is not guaranteed or endorsed by the publisher.

## Footnotes

1
http://dbm.neuro.uni-jena.de/cat/

2
http://www.fil.ion.ucl.ac.uk/spm/

## Abbreviations

AD: Alzheimer’s disease
SD: semantic dementia
PCA: posterior cortical atrophy
lvPPA: logopenic variants primary progressive aphasia
nfvPPA: non-fluent/agrammatic variants primary progressive aphasia
ATL: anterior temporal lobe
MRI: magnetic resonance imaging
FDG: 2-fluoro-2-deoxy-D-glucose
PET: positron emission tomography
CSF: cerebrospinal fluid
SUVR: standard uptake value ratio
SPECT: single-photon emission computed tomography
HC: healthy control
MMSE: Mini-Mental State Exam
MoCA: Montreal Cognitive Assessment
CDR: clinical dementia rate
CDT: Clock Drawing Test
BNT: Boston Naming Test
TE: echo time
TR: repetition time
TI: inversion time
rsfMRI: resting-state functional MRI
GM: gray matter
WM: white matter
FC: functional connectivity.
