# Supplementary material for: Characterizing Differences in Functional Connectivity Between Posterior Cortical Atrophy and Semantic Dementia by Seed-Based Approach
Source: Front Aging Neurosci. 2022 Apr 29;14:850977. doi: 10.3389/fnagi.2022.850977 (PMC9099291; doi:10.3389/fnagi.2022.850977)
Supplement: Supplementary file 1 [file Data_Sheet_1.docx]

**Supplementary Tables and Figures**

**Supplementary Table 1.** Partial correlation analysis between Functional connectivity intensity and neuropsychological scales (only patient group)

| **Functional connectivity changing groups** | **Clusters (seeds)** | **Neuropsychological scales** | | | |
| --- | --- | --- | --- | --- | --- |
|  |  | **MMSE** | **MoCA** | **BST** | **CDT** |
| PCA compared to NC  (without NC) | Right medial frontal gyrus (left anterior temporal lobe)  PCA>NC | r = -0.342, p = 0.508 | r = -0.919, p = 0.001 | r = 0.113, p = 0.831 | r = -0.492, p = 0.322 |
|  | Left superior temporal gyrus (left anterior insula)  PCA>NC | r = 0.177, p = 0.737 | r = -0.116, p = 0.827 | r = -0.902, p = 0.014 | r = -0.682, p = 0.136 |
|  | Left fusiform (left superior temporal gyrus)  PCA>NC | r = -0.309, p = 0.551 | r = 0.315, p = 0.544 | r = 0.230, p = 0.661 | r = 0.088, p = 0.868 |
|  | Left precentral areas (left V1)  PCA<NC | r = 0.382, p = 0.455 | r = 0.297, p = 0.567 | r = 0.444, p = 0.378 | r = 0.790, p = 0.061 |
|  | Right inferior frontal gyrus (right V1)  PCA>NC | r = 0.109, p = 0.837 | r = -0.636, p = 0.174 | r = -0.372, p = 0.526 | r = -0.473, p = 0.344 |
| SD compared to NC  (without NC) | Right superior frontal gyrus (left anterior temporal lobe)  SD<NC | r = 0.283, p = 0.498 | r = 0.277, p = 0.507 | r = 0.294, p = 0.479 | r = 0.149, p = 0.725 |
|  | Right superior temporal gyrus (left anterior temporal lobe)  SD>NC | r = 0.097, p = 0.820 | r = 0.105, p = 0.805 | r = -0.163, p = 0.699 | r = 0.144, p = 0.734 |
|  | Right insula (right anterior insula)  SD>NC | r = -0.126, p = 0.766 | r = -0.047, p = 0.912 | r = -0.146, p = 0.730 | r = -0.166, p = 0.692 |
|  | Left superior frontal gyrus (right anterior insula)  SD<NC | r = -0.058, p = 0.892 | r = -0.164, p = 0.697 | r = 0.331, p = 0.423 | r = -0.166, p = 0.695 |
|  | Left anterior cingulum (right anterior insula)  SD>NC | r = 0.159, p = 0.77 | r = 0.184, p = 0.663 | r = 0.138, p = 0.745 | r = 0.046, p = 0.914 |
|  | Right middle frontal gyrus (left superior temporal gyrus)  SD<NC | r = -0.275, p = 0.509 | r = -0.382, p = 0.351 | r = 0.237, p = 0.573 | r = -0.395, p = 0.332 |
|  | Right superior frontal gyrus (left superior temporal gyrus)  SD<NC | r = 0.439, p = 0.277 | r = 0.417, p = 0.304 | r = 0.674, p = 0.067 | r = 0.338, p = 0.413 |

NC, normal control; PCA, posterior cortical atrophy; SD, semantic dementia; MMSE, Mini-Mental State Examination; MoCA, Montreal Cognitive Assessment; BNT, Boston Naming Test; CDT, Clock Drawing Test.

**Supplementary Table 2.** The summary of the FC change, FC-cognition relation, and PET change in PCA and SD.

|  | **PCA** | **SD** |
| --- | --- | --- |
| FC change | Both increased and decreased regional FC were showed with seeds in visual, language and salience network. The region with abnormal FC could spread to parietal areas. | Both increased and decreased regional FC were showed with seeds in language and salience network, and more anterior regions were affected. The region with abnormal FC was restricted in frontal-temporal areas. |
| FC-cognition relation | The relations were significant when controls are included. Removing the controls, few relations were significant. | The relations were significant when controls are included. Removing the controls, no relation was significant. |
| PET change | The regions with abnormal FC were almost accompanied by hypometabolism. | The most regions with abnormal FC were accompanied by hypometabolism. |

PCA, posterior cortical atrophy; SD, semantic dementia; FC, functional connectivity; PET, positron emission tomography.


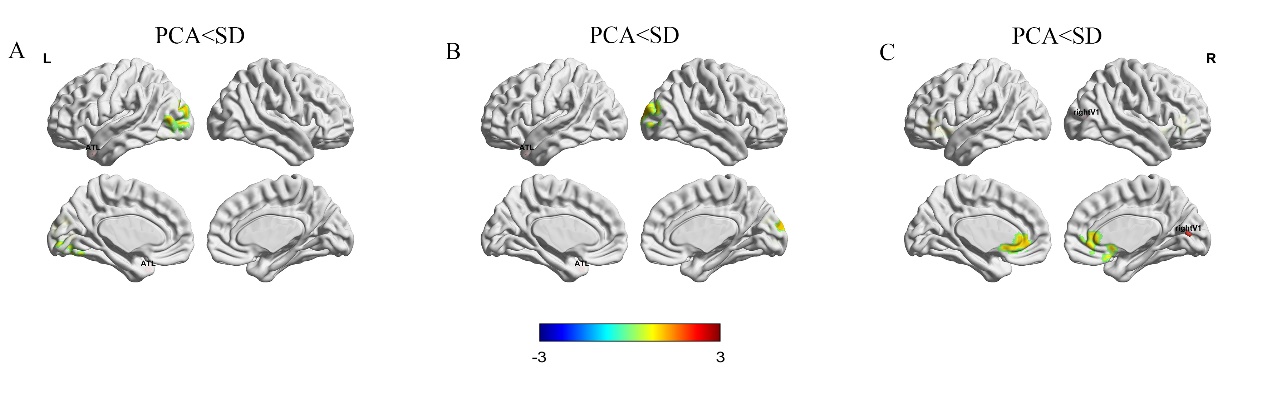


**Supplementary Figure 1**. Group differences in regional functional connectivity from seed between PCA and SD. The results were mapped on the brain surface using BrainNet Viewer (Xia et al.,2013) showing changed functional connectivity from seeds (A: left anterior temporal lobe, B: left anterior temporal lobe, C: right V1) to bilateral middle occipital lobe (A and B) and bilateral anterior cingulum (C). The red spheres were localization of seeds. Colors indicate t scores. ATL, anterior temporal lobe.


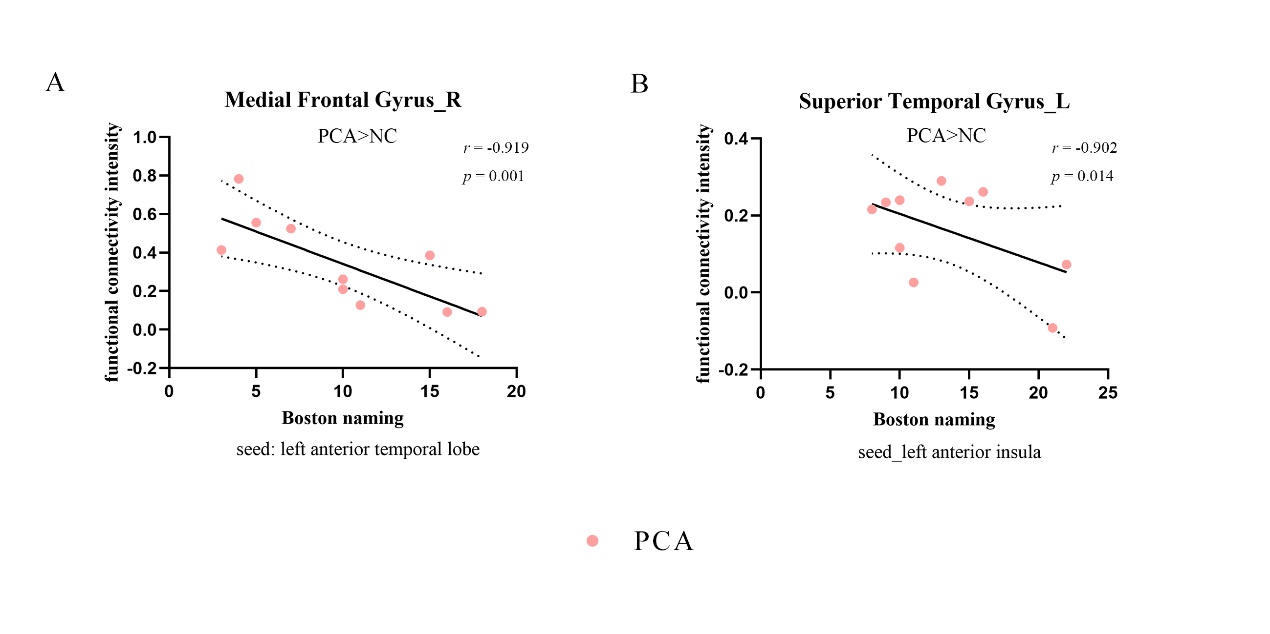


**Supplementary Figure 2**. Correlation between functional connectivity and subdomain of cognition in PCA patients. The scatterplots illustrate the associations between MoCA (A) or BST (B) scores and intensity of functional connectivity in PCA patients.


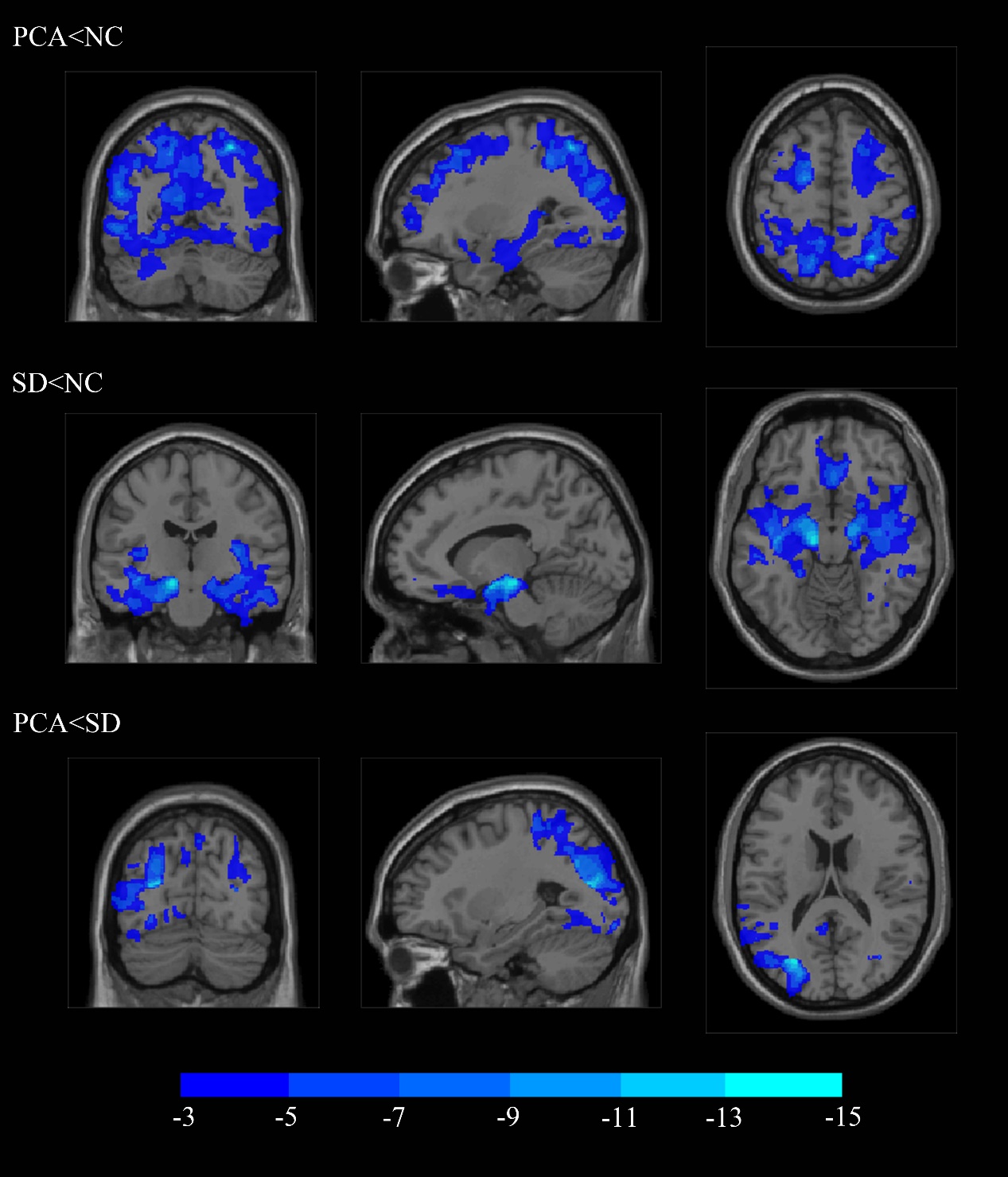


**Supplementary Figure 3**. The atrophy pattern of patients. A: The atrophy of PCA compared to NC; B: The atrophy of SD compared to NC; C: The atrophy of PCA compared to SD. Colors indicate t scores.


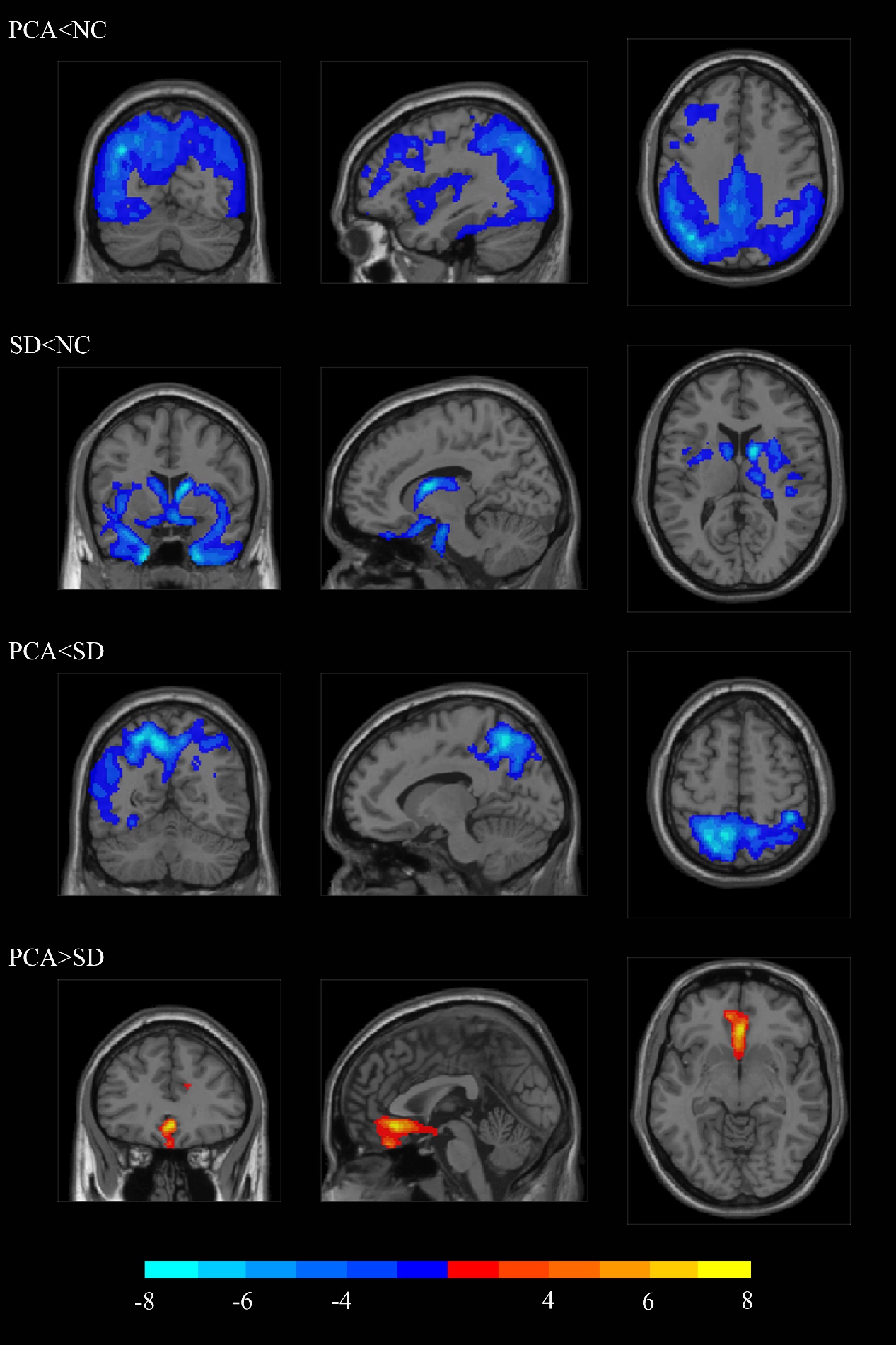


**Supplementary Figure 4**. The hypometabolism pattern of patients. A: The hypometabolism of PCA compared to NC; B: The hypometabolism of SD compared to NC; C&D: The hypometabolism of PCA compared to SD. Colors indicate t scores.
